# Efficacy and safety of tranexamic acid administration in traumatic brain injury patients: a systematic review and meta-analysis

**DOI:** 10.1186/s40560-020-00460-5

**Published:** 2020-07-03

**Authors:** Shoji Yokobori, Tomoaki Yatabe, Yutaka Kondo, Kosaku Kinoshita, for the Japan Resuscitation Council (JRC) Neuroresuscitation Task Force and the Guidelines Editorial Committee, Yasuhiko Ajimi, Masaaki Iwase, Kyoko Unemoto, Junji Kumasawa, Jun Goto, Hitoshi Kobata, Atsushi Sawamura, Toru Hifumi, Eisei Hoshiyama, Mitsuru Honda, Yasuhiro Norisue, Shoji Matsumoto, Yasufumi Miyake, Takashi Moriya, Hideto Yasuda, Kazuma Yamakawa, Sunghoon Yang, Masahiro Wakasugi, Masao Nagayama, Hiroshi Nonogi

**Affiliations:** 1 Department of Emergency and Critical Care Medicine, Nippon Medical School, 1-1-5 Sendagi, Bunkyo-Ku, Tokyo, 113-8603 Japan; 2 Department of Anesthesiology and Intensive Care Medicine, Kochi Medical School, Kochi, Japan; 3 Department of Emergency and Critical Care Medicine, Juntendo University Urayasu Hospital, Chiba, Japan; 4 Department of Acute Medicine, Nihon University School of Medicine, Tokyo, Japan

**Keywords:** Clotting, TBI, Head-trauma, Hemorrhage, Fibrinolysis, Hematoma, Meta-analysis

## Abstract

**Background:**

The exacerbation of intracranial bleeding is critical in traumatic brain injury (TBI) patients. Tranexamic acid (TXA) has been used to improve outcomes in TBI patient. However, the effectiveness of TXA treatment remains unclear. This study aimed to assess the effect of administration of TXA on clinical outcomes in patients with TBI by systematically reviewing the literature and synthesizing evidence of randomized controlled trials (RCTs).

**Methods:**

MEDLINE, the Cochrane Central Register of Controlled Trials, and Igaku Chuo Zasshi (ICHUSHI) Web were searched. Selection criteria included randomized controlled trials with clinical outcomes of adult TBI patients administered TXA or placebo within 24 h after admission. Two investigators independently screened citations and conducted data extraction. The primary “critical” outcome was all-cause mortality. The secondary “important” outcomes were good neurological outcome rates, enlargement of bleeding, incidence of ischemia, and hemorrhagic intracranial complications. Random effect estimators with weights calculated by the inverse variance method were used to report risk ratios (RRs).

**Results:**

A total of 640 records were screened. Seven studies were included for quantitative analysis. Of 10,044 patients from seven of the included studies, 5076 were randomly assigned to the TXA treatment group, and 4968 were assigned to placebo. In the TXA treatment group, 914 patients (18.0%) died, while 961 patients (19.3%) died in the placebo group. There was no significant difference between groups (RR, 0.93; 95% confidence interval, 0.86–1.01). No significant differences between the groups in other important outcomes were also observed.

**Conclusions:**

TXA treatment demonstrated a tendency to reduce head trauma-related deaths in the TBI population, with no significant incidence of thromboembolic events. TXA treatment may therefore be suggested in the initial TBI care.

## Background

Traumatic brain injury (TBI) is a significant cause of mortality and morbidity worldwide, especially in children and young adults. In Japan, the main victims are elderly people, which exhibit slow recovery and therefore incur high medical costs [1]. TBI causes a high socioeconomic burden, incurring high medical expenses and loss of productivity [1, 2]. To reduce undesired outcomes of TBI, efforts to develop TBI treatment have been performed. However, the prognosis of TBI remains poor. In the USA, approximately 50,000 people die and at least 5.3 million live with disabilities related to TBI per year [3].

Recently, the pathophysiology of coagulopathy has been the focus of trauma care [4, 5]. Immediately after having TBI, the state of hyperfibrinolysis peaks within 3 h which causes hematoma expansion [6]. Thus, early (< 1 h and no later than 3 h after injury) treatment with tranexamic acid (TXA), an anti-fibrinolysis drug, may be ideal for this trauma population [7, 8].

Several randomized controlled trials (RCTs) examining the efficacy of TXA in trauma patients have been performed in recent years [9–11]. However, an in-depth meta-analysis of the latest results of these larger RCTs is lacking. The aim of this study was thus to clarify the efficacy of acute TXA treatment in TBI patients by analyzing recent literatures.

## Methods

We organized the systematic review team in the Japan Resuscitation Council (JRC) Neuroresuscitation Task Force. The JRC Neuroresuscitation Task Force and the Guidelines Editorial Committee was established in 2020 which organized by the Japan Society of Neuroemergencies and Critical Care, the Japanese Society of Intensive Care Medicine, and the Japan Society of Neurosurgical Emergency. The JRC Neuroresuscitation Task Force sets six clinically relevant questions, and this systematic review was performed.

We conducted a systematic review that conformed to the Preferred Reporting Items for Systematic Reviews and Meta-Analyses (PRISMA) standards [12]. This study was also registered in the University Hospital Medical Information Network (UMIN) Clinical Trials Registry, which is the largest clinical trial registry in Japan (UMIN ID, 000040389).

With the discussion in this JRC Neuroresuscitation Task Force, population intervention comparator outcome study design and timeframe (PICOST) to guide a systematic review search was set as below.

P (patients): All types of adult TBIs.

I (interventions): Initial administration of TXA within 24 h after injury. Dose and method of administration of TXA were not limited.

C (comparisons, controls): Placebo or non-intervention.

O (outcomes): Primary, “critical” outcome as mortality from any cause and secondary, “important” outcomes as poor neurological outcomes (severe disability, vegetable state, and death in Glasgow Outcome Scale), ischemic or thromboembolic complications, and hemorrhagic complications.

S (study design): RCTs.

T (timeframe): All publisted literatures up to October 26, 2019.

We identified RCTs investigating the effects of TXA on mortality in TBI patients by searching PubMed, the Cochrane library, and Igaku Chuo Zasshi (ICHUSHI) Web up to October 26, 2019. ICHUSHI Web is the largest database of Japanese medical journals, containing approximately 10 million manuscripts from 6000 journals.

We included studies that fulfilled the following criteria: (1) an RCT, (2) a full-text publication in English or Japanese, (3) included adult patients with TBI, (4) included comparisons between TXA and placebo or non-intervention, and (5) initial administration of TXA within 24 h after injury. Dose and method of administration of TXA were not limited.

Two reviewers (TY and SY) independently abstracted the data and assessed the methodologic quality of the eligible studies. Two reviewers also achieved the consensus on this literature selection, and any disagreement between them over the eligibility of particular studies was resolved through discussion. Data abstracted from each study included the first author’s name, year of publication, number of study sites, number of patients, patient ages, proportion of females, duration between injury and administration of TXA, dose of TXA, and other treatment (surgery, transfusion, etc.). Methodologic quality was evaluated using the Cochrane risk of bias assessment tool [13], which assesses randomization; allocation concealment; blinding of study participants, personnel, and outcome assessments; incomplete outcome data; selective outcome reporting; and other potential sources of bias. The two reviewers achieved the consensus on the risk of bias (RoB), and any discrepancy of judge was resolved through discussion.

The grades of recommendation, assessment, development, and evaluation (GRADE) approach was also used to evaluate the certainty of the available evidence, like as inconsistency, indirectness, imprecision, and publication bias. We provided the evidence profile table using the GRADE pro GDT (GRADEpro GDT: GRADEpro Guideline Development Tool [Software]. McMaster University, 2015 (developed by Evidence Prime, Inc.). Available from gradepro.org.). For the application of GRADE system, we received guidance from the Medical Information Network Distribution Service (MINDS), a Japanese center for GRADE education. Two reviewers also discussed the results of risk of bias and achieved the consensus on this final decision.

According to this GRADE approach, primary outcome was replaced as “critical,” and secondary outcomes were replaced as “important” outcomes [14].

We defined the critical outcome as hospital mortality and important outcomes as favorable neurological outcomes, progressive intracranial hemorrhage, and complications (thrombosis and bleeding). If hospital mortality data were not available, we substituted the 28 to 90-day mortality for hospital mortality. Poor neurological outcome was defined as severe disability (SD), persistent vegetable state (PVS), and dead (D) on Glasgow Coma Scale.

We performed the meta-analysis using Review Manager, version 5.3 (The Nordic Cochrane Centre, The Cochrane Collaboration, Copenhagen, Denmark). Comparative odds ratios (ORs) were reported with their associated 95% confidence intervals (CIs). We selected a random effects model. Statistical heterogeneity was determined by assessing *I*^2^ values, which were interpreted as follows: 0–40%, might not be important; 30–60%, may represent moderate heterogeneity; 50–90%, may represent substantial heterogeneity; and 75–100%, may represent considerable heterogeneity.

We conducted subgroup analyses to evaluate each outcome using only low risk of RoB RCTs. Therefore, we decided to create the evidence profile table based on this subgroup analyses before starting systematic review.

## Results

### Literature search strategy

**Fig. 1:**
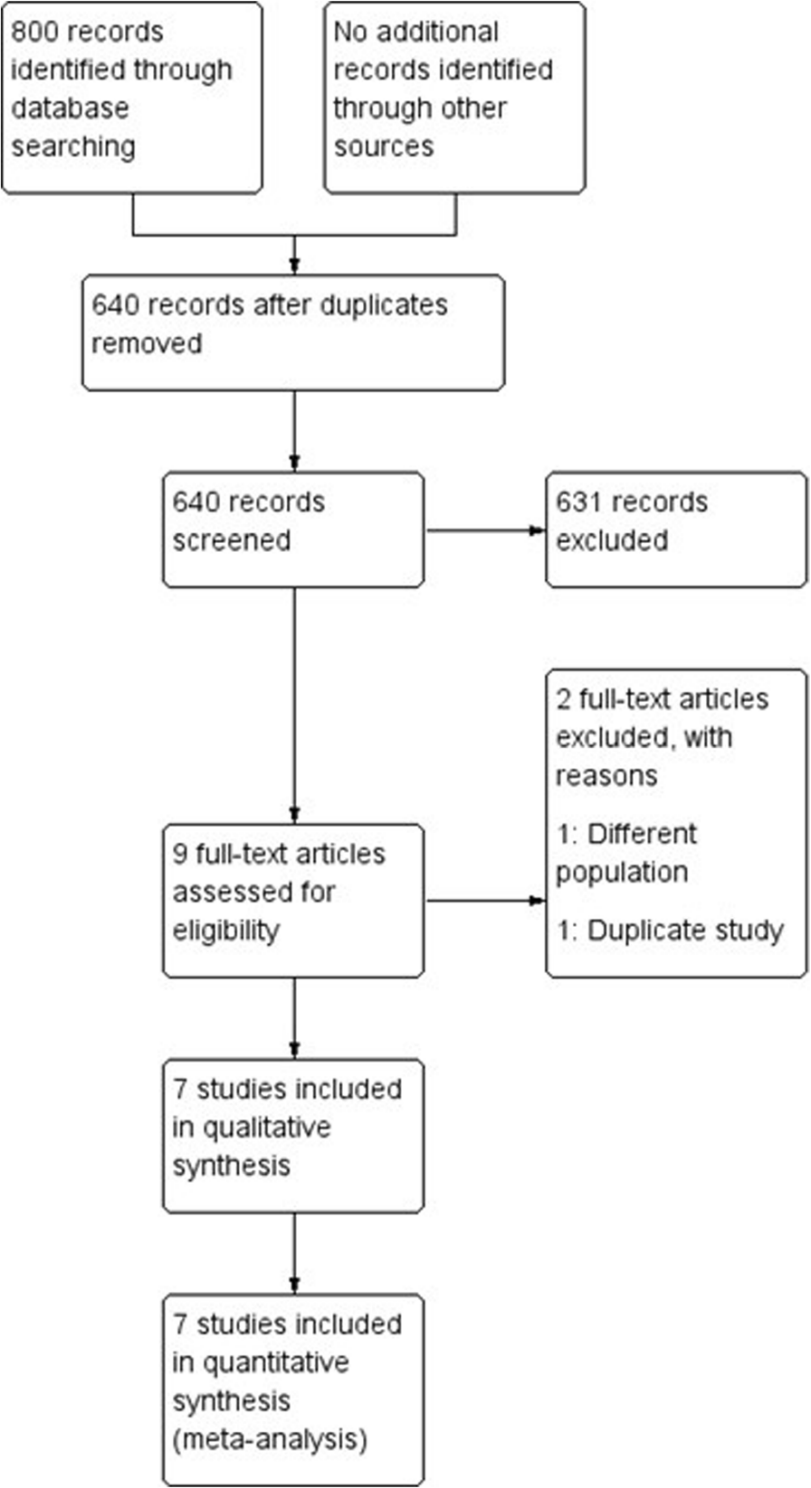
Flow chart of the search strategy and study selection

A total of 800 studies were identified through database searching. After removing duplicated literature, 640 studies were eligible. Based on title and abstract assessment, 631 study records were excluded, and nine full text articles were included for full-text article assessment. After reading the full study literature, one study was excluded due to different patient populations, and one study was excluded due to study duplication. Thus, seven RCTs were finally included in this meta-analysis (Fig. 1). Searching formulae and the results of search are presented as Supplementary Table 1.

### Characteristics

Characteristics of the included studies are summarized in Table 1. A total of 10,124 patients selected from seven RCTs [9, 15–18] were randomly assigned to the TXA treatment group (*n* = 5116) versus placebo (*n* = 5,008) for critical outcome analysis. Each study included 80 to 9127 patients, with ages ranging from 32 to 42 years. The proportion of women was 9 to 25%. The largest RCT population was in the CRASH-3 study, which was published in 2019 [9]. Two studies were multicenter RCTs. In all studies, initial dose of TXA was 1 g, and maintenance dose was 1 g.

**Table 1:** Characteristics of included randomized control trials

Author, year	No. of sites	No. of patients	Age (years)	Female (%)	Traffic accident (%)	Polytrauma (%)	GCS on arrival	ISS	Initial TXA	Maintenance TXA	sBP (mmHg)	Initial hemorrhage volume (mL)
Chakroun-Walha et al. 2018 [15]	1	180	41 ± 19	9	91	57	9 vs 10	22 vs 24	1 g/10 min	1 g/8 h	Mean BP 87 vs 89	N/A
Fakharian et al. 2018 [16]	1	149	42 vs 39	11	85	N/A	13 vs 12	N/A	1 g/10 min	1 g/8 h	118 vs 120	N/A
Jokar et al. 2017	1	80	35 vs 36	25	21	N/A	N/A	N/A	1 g/10 min	1 g/8 h	160/162	22 vs 22
Yutthakasemsunt et al. 2013 [17]	1	238	35 vs 34	12	N/A	85	12 and under 42%	23 vs 25	1 g/30 min	1 g/8 h	N/A	N/A
CRASH-2 2010 [10]	10	270	36 vs 37	15	N/A	N/A	12 and under 53%	N/A	1 g/10 min	1 g/8 h	Less than 90 mmHg 7%	17 vs 20
CRASH-3 2019 [9]	175	9202	42 vs 42	20	N/A	N/A	12 and under 71%	N/A	1 g/10 min	1 g/8 h	Less than 90 mmHg 2%	N/A
Ebrahimi et al. 2019 [18]	1	80	32 vs 33	15	N/A	N/A	12 and under 68%	N/A	1 g/10 min	1 g/8 h	N/A	N/A

*No.* number, *GCS* Glasgow Coma Scale, *TXA* tranexamic acid, *sBP* systolic blood pressure, *N/A* not available, *ISS* Injury Severity Score, *CRASH-2* CRASH-2 Collaborators, Intracranial Bleeding Study

### Critical outcomes

**Fig. 2:**
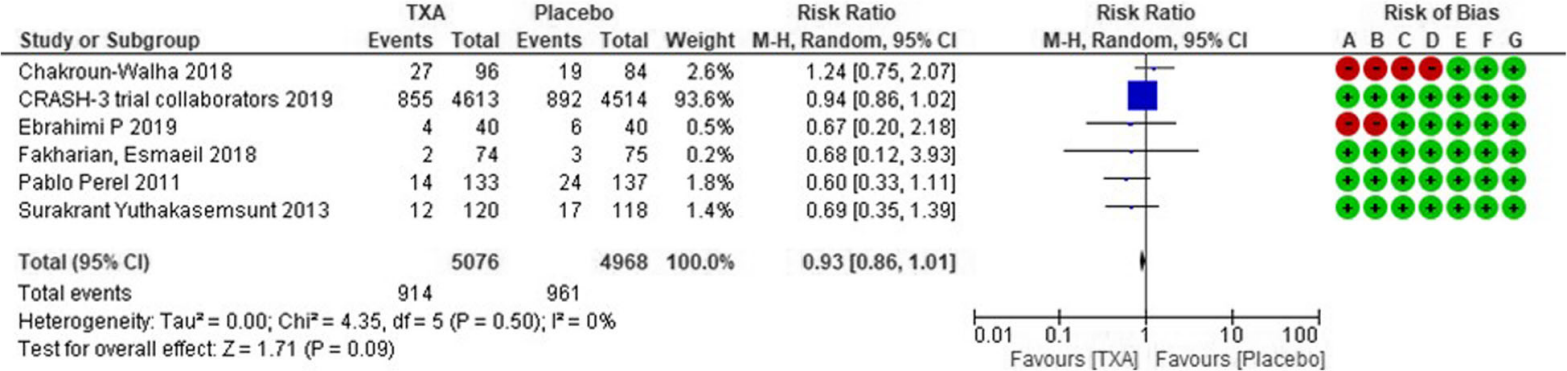
Forest plot comparing the all-cause mortality values between the tranexamic acid and placebo groups. Risk of bias summary is listed as follows: **a**, random sequence generation (selection bias); **b**, allocation concealment (selection bias); **c**, blinding of participants and personnel (performance bias); **d**, blinding of outcome assessment (detection bias); **e**, incomplete outcome data (attrition bias); **f**, selective reporting (reporting bias); and **g**, other bias. TXA, tranexamic acid; CI, confidence interval

Mortality from any cause was evaluated in six RCTs. Evidence profiles were shown as Supplementary Table 2. This set of six RCTs had less publication bias with the symmetric distribution in funnel plot (Supplementary Figure). With these RCTs analyzed, the forest plot of the critical outcomes is shown in Fig. 2. In this observation period, 914 patients (18.0%) died in the TXA-treated group, while 961 patients (19.3%) died in the placebo control group. There was a trend for superior critical outcomes in the TXA-treated group (RR, 0.93 [95% CI, 0.85–1.01]); however, this did not reach significance (*P* = 0.09, Fig. 2).

### Important outcomes

**Fig. 3:**
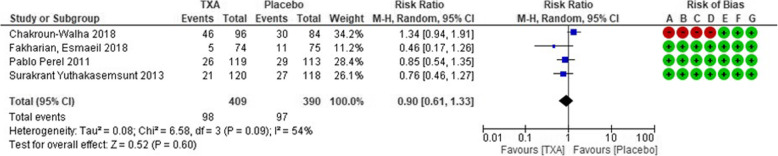
Forest plot comparing poor neurological outcome rates between the tranexamic acid and placebo groups. Risk of bias summary is listed as follows: **a**, random sequence generation (selection bias); **b**, allocation concealment (selection bias); **c**, blinding of participants and personnel (performance bias); **d**, blinding of outcome assessment (detection bias); **e**, incomplete outcome data (attrition bias); **f**, selective reporting (reporting bias); and **g**, other bias. TXA, tranexamic acid; CI, confidence interval

The incidence of poor neurological outcomes was clarified in four RCTs [10, 15–17]. With this study population (*n* = 799), the forest plot for the detection of poor outcomes is shown in Fig. 3. Of 409 patients, 98 (23.9%) which received TXA exhibited poor outcomes, while 97 of 390 patients (24.9%) exhibited poor outcomes in the placebo control group. There was no significant difference in incidence of poor outcomes between TXA and placebo (RR, 0.90; 95% CI, [0.61–1.33]; *P* = 0.60).

**Fig. 4:**
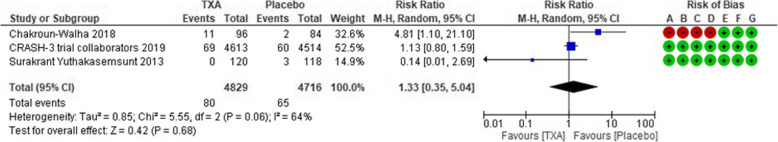
Forest plot comparing the incidence of ischemic or thromboembolic complications between the tranexamic acid and placebo-control groups. Risk of bias summary is listed as follows: **a**, random sequence generation (selection bias); **b**, allocation concealment (selection bias); **c**, blinding of participants and personnel (performance bias); **d**, blinding of outcome assessment (detection bias); **e**, incomplete outcome data (attrition bias); **f**, selective reporting (reporting bias); and **g**, other bias. TXA, tranexamic acid; CI, confidence interval

**Fig. 5:**
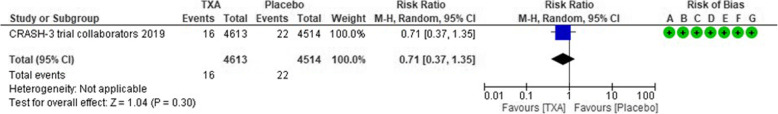
Forest plot comparing the incidence of hemorrhagic complications between the tranexamic acid and control groups. Risk of bias summary is listed as follows: **a**, random sequence generation (selection bias); **b**, allocation concealment (selection bias); **c**, blinding of participants and personnel (performance bias); **d**, blinding of outcome assessment (detection bias); **e**, incomplete outcome data (attrition bias); **f**, selective reporting (reporting bias); and **g**, other bias. TXA, tranexamic acid; CI, confidence interval

Three RCTs [9, 15, 17] reported the incidence of ischemic complication, and only one RCT [9] reported hemorrhagic complication. With these patient cohorts (*n* = 9545 for ischemic complications and *n* = 9127 for hemorrhagic complications), sub-analyses did not reveal any significant differences (ischemic complication: RR, 1.33; 95% CI, [0.35–5.04]; *P* = 0.68; hemorrhagic complication: RR, 0.71; 95% CI, [0.37–1.35]; *P* = 0.30; Figs. 3, 4, and 5, respectively).

### Subgroup analysis using low RoB RCTs

To assure the strong evidence on these critical and important outcomes, we chose low RoB RCSs to create the evidence profile table (Table 2). In this sub-analysis, four low RCTs are selected for the analysis of primary critical outcome, and there was a non-significant trend for superior in the TXA-treatment group (RR, 0.93 [95% CI, 0.85–1.01]), as same as primary analysis with 6 RCTs. Sub-analyses with low RoB relating on the three important outcomes also did not reveal any significant differences (poor neurological outcome: RR, 0.76 95% CI, [0.55–1.06]; ischemic complication: RR, 0.68; 95% CI, [0.12–3.93]; hemorrhagic complication: RR, 0.71; 95% CI, [0.37–1.35]) (Table 2).

**Table 2:** Evidence profile

Certainty assessment							No. of patients		Effect		Certainty	Importance
No. of studies	Study design	Risk of bias	Inconsistency	Indirectness	Imprecision	Other considerations	TXA	Placebo	Relative (95% CI)	Absolute (95% CI)		
Mortality from any cause (low RoB)												
4	Randomized trials	Not serious	Not serious	Not serious	Not serious	None	883/4940 (17.9%)	936/4844 (19.3%)	RR 0.93 (0.85 to 1.01)	14 fewer per 1000 (from 29 fewer to 2 more)	High ⊕⊕⊕⊕	Critical
Poor neurological outcome (low RoB)												
3	Randomized trials	Not serious	Not serious	Not serious	Serious^a^	None	52/313 (16.6%)	67/306 (21.9%)	RR 0.76 (0.55 to 1.06)	53 fewer per 1000 (from 99 fewer to 13 more)	Moderate ⊕⊕⊕〇	Critical
Ischemic or thromboembolic complications (low RoB)												
2	Randomized trials	Not serious	Serious^b^	Not serious	Very serious^c^	None	69/4733 (1.5%)	63/4632 (1.4%)	RR 0.68 (0.12 to 3.93)	4 fewer per 1000 (from 12 fewer to 40 more)	Very low ⊕〇〇〇	Critical
Hemorrhagic complications (bleeding, low RoB)												
1	Randomized trials	Not serious	Not serious	Not serious	Very serious^c^	None	16/4613 (0.3%)	22/4514 (0.5%)	RR 0.71 (0.37 to 1.35)	1 fewer per 1000 (from 3 fewer to 2 more)	Low ⊕⊕〇〇	Critical

*CI* confidence interval, *RR* risk ratio, *RoB* risk of bias

Reasons of downgrade are as follows:

^a^Sample size is smaller than optimal information size. In addition, 95% CI is wide

^b^*I*^2^ value is high

^c^Sample size is smaller than optimal information size. In addition, 95% CI is very wide

## Discussion

In this systematic review and meta-analysis, we aimed to clarify the efficacy of TXA administration compared to that of placebo in TBI patients. Several systematic reviews of TXA treatment in TBI patients have been published [19–21]. However, in these meta-analyses, the largest RCT, CRASH-3 Trial [9], was not included in the literature search. Our review is thus the first largest (*n* = 10,044) systematic review and meta-analysis of RCTs to compare TXA treatment and placebo. Our meta-analysis revealed that TXA administration showed a tendency to reduce head trauma-related death, but the results were not statistically significant.

TXA is an anti-fibrinolytic agent which has been used to treat or prevent excessive blood loss with many medical and surgical indications [22], including major trauma [8], postpartum bleeding [23], and orthopedic surgery [24]. TXA is a synthetic analog of the lysine, a kind of amino acid. This reduces conversion of plasminogen to plasmin, preventing fibrin degradation and preserving the framework of fibrin’s matrix structure. Thus, TXA serves as an antifibrinolytic agent. Indications for TXA treatment have been investigated clinically, targeting characteristic pathophysiology in TBI.

TBI is associated with coagulopathy, and the background of this pathophysiology has been reported in the recent literature [6, 25]. The pathophysiology of coagulopathy in TBI includes tissue factor activation, thrombocytopenia, platelet dysfunction, protein C activation, and hyperfibrinolysis. The main mechanism of TBI-related coagulopathy is hyperfibrinolysis followed by primary consumptive coagulopathy that is caused by migration of tissue factors from injured brain tissue to blood. The peak of this hyperfibrinolysis occurs approximately 3 h after experiencing the injury [6]. Early treatment with TXA, an-anti fibrinolytic agent, is therefore considered reasonable.

Previous clinical research supports this hypothesis. The recent CRASH-2 trial, in which 20,211 trauma patients in 40 countries were enrolled, demonstrated the efficacy of TXA treatment for reducing bleeding in trauma patients [10] and reducing mortality in perinatal hemorrhagic patients [23].

Recently, CRASH-3 Trial, an RCT using TXA treatment for TBI, reported that TXA treatment was safe in patients with TBI. Among patients treated within 3 h of injury, the risk of head injury-related deaths was lower (18.5%) in the TXA group compared to 19.8% in the placebo (RR, 0.94; 95% CI, 0.86–1.02) in primary intention-to-treat analysis, but the superiority of TXA was not statistically significant [9]. Also, the risk of head injury-related death did not reduced with tranexamic acid in patients with severe head injury [9]. Thus, we aimed to establish this systematic review to clarify whether TXA treatment was effective for TBI patients by analyzing large patient cohorts. The objective of this systematic review was to address the following research question: in TBI patients entering the emergency room with or being at risk of TBI (patients), does administration of TXA (intervention) compared to placebo (comparison) improves patients’ outcomes such as reduction in mortality, neurological function, and hemorrhage/ischemia progression (outcome).

Our analysis of 10,124 patients from seven RCTs revealed a trend for efficacy in the TXA treatment group (mortality in all TXA-treated patients, 18.0%) versus the placebo-control (normal saline) group (mortality, 19.3%), although this difference was not statistically significant.

CRASH-3 demonstrated that the risk of head injury-related deaths was reduced with TXA treatment in patients with mild-to-moderate head injury (RR, 0.78; 95% CI, 0.64–0.95) but not in patients with severe head injury (RR, 0.99; 95% CI, 0.91–1.07; *p* = 0.73), suggesting that TXA may be more effective in non-severe TBI patients. Further, CRASH-3 concluded that TXA treatment was more effective than later treatment in patients with mild and moderate head injury. It is necessary to understand the exact indication of TXA (i.e., severity and type of TBI) to determine which patients will benefit from TXA treatment.

Our results highlighted the safety and feasibility of TXA treatment with no significant difference in incidence of thromboembolic complications (1.7% in TXA versus 1.4% in placebo group; RR, 1.33; 95% CI, 0.35–5.04; *p* = 0.68). This result warrants the use of TXA for TBI, even in non-severe TBI. TXA is an inexpensive drug and is listed as an essential drug by the World Health Organization [26]. Even in developing countries, TXA treatment may be feasible in daily TBI care.

This meta-analysis has several limitations. First, only seven RCTs were included in our systematic review, with limited information on complications such as seizures [27]. Second, the severity and type of TBI were not mentioned in included RCTs. For example, the patient inclusion criteria of CRASH-3 were (1) Glasgow Coma Scale (GCS) score of 12 or lower and (2) intracranial bleeding on CT scan and no major extracranial bleeding. Thus, in the CRASH-3 trial, the pathophysiology of concussion or diffuse axonal injury was not completely ruled out. To titrate the indication of TXA for TBI, well-designed RCTs are needed in the future to support our findings.

Only one RCT (CRASH-3) mentioned hemorrhagic complications including hemorrhage worsening or progression. Further RCTs are required to form definite conclusions regarding the robustness/safety of this treatment.

## Conclusions

Our meta-analysis demonstrated that TXA treatment showed a trend for reducing head trauma-related death in TBI patients, with no significant incidence of thromboembolic events. TXA treatment may therefore be suggested in the initial TBI care.

## Supplementary information

**Additional file 1: Supplementary figure.** Funnel plot of 6 randomized controlled trials. This set of six RCTs had less publication bias with the symmetric distribution.

**Additional file 2: Supplementary Table 1.**

**Additional file 2: Supplementary Table 2.** Evidence profile (Including all RoB). Reasons of downgrade: a) Sample size is smaller than optimal information size. In addition, 95% CI is wide. b) I2 value is high. c) Sample size is smaller than optimal information size. In addition, 95% CI is very wide. CI: Confidence interval; RR: Risk ratio; RoB: Risk of Bias.

## Abbreviations

TBI: Traumatic brain injury
TXA: Tranexamic acid
RR: Risk ratio
RCT: Randomized controlled trial
