# Supplementary figures and images for: Efficacy and safety of tranexamic acid administration in traumatic brain injury patients: a systematic review and meta-analysis
Source: J Intensive Care. 2020 Jul 3;8:46. doi: 10.1186/s40560-020-00460-5 (PMC7333334; doi:10.1186/s40560-020-00460-5)

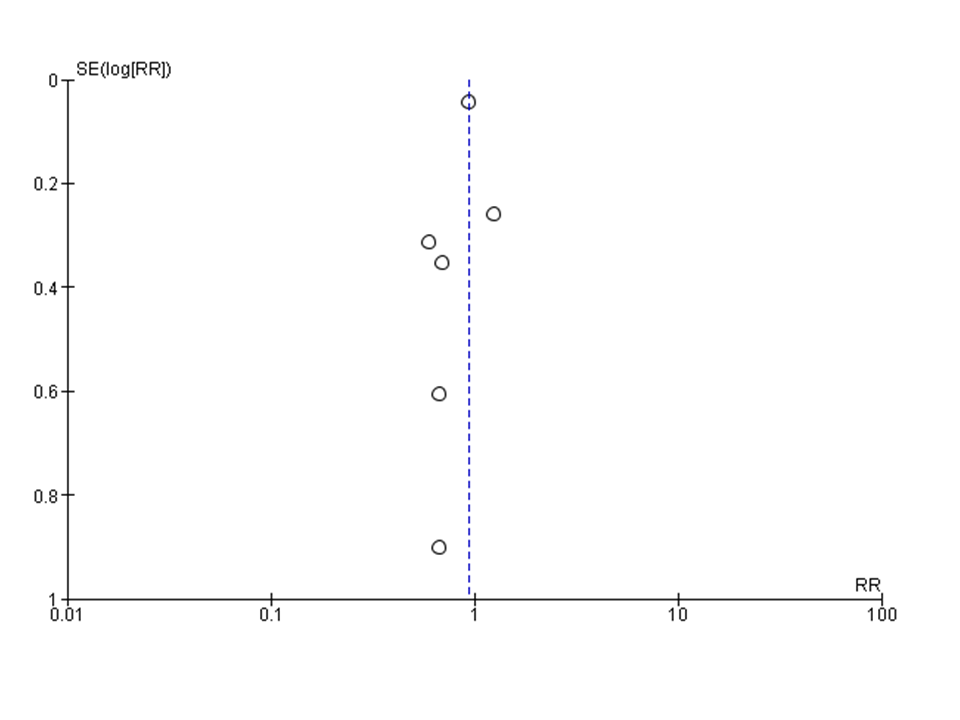

Supplement: Supplementary file 1 — Additional file 1: Supplementary figure. Funnel plot of 6 randomized controlled trials. This set of six RCTs had less publication bias with the symmetric distribution. [file 40560_2020_460_MOESM1_ESM.tif]
