# Supplementary material for: Efficacy and safety of tranexamic acid administration in traumatic brain injury patients: a systematic review and meta-analysis
Source: J Intensive Care. 2020 Jul 3;8:46. doi: 10.1186/s40560-020-00460-5 (PMC7333334; doi:10.1186/s40560-020-00460-5)
Supplement: Supplementary file 2 — Additional file 2: Supplementary Table 1. [file 40560_2020_460_MOESM2_ESM.docx]

| Certainty assessment | | | | | | | No. of patients | | Effect | | Certainty | Importance |
| --- | --- | --- | --- | --- | --- | --- | --- | --- | --- | --- | --- | --- |
| No. of studies | Study design | Risk of bias | Inconsistency | Indirectness | Imprecision | Other considerations | TXA | Placebo | Relative (95% CI) | Absolute  (95% CI) |  |  |
| Mortality | | | | | | | | | | | | |
| 6 | Randomized trials | Serious | Not serious | Not serious | Not serious | None | 914/5076  (18.0%) | 961/4968  (19.3%) | RR 0.93  （0.86 to 1.01） | 14 fewer per 1,000  (from 27 fewer to 2 more) | Moderate  ⊕⊕⊕〇 | Critical |
| Poor neurological outcome | | | | | | | | | | | | |
| 4 | Randomized trials | Serious | Serious | Not serious | Serious^a^ | None | 98/409  (24.0%) | 97/390  (24.9%) | RR 0.90  （0.61 to 1.33） | 25 fewer per 1,000  (from 97 fewer to 82 more) | Very low  ⊕〇〇〇 | Critical |
| Complication (Ischemia and thrombosis) | | | | | | | | | | | | |
| 3 | Randomized trials | Serious | Serious^b^ | Not serious | Very Serious^c^ | None | 80/4829  (1.7%) | 65/4716  (1.4%) | RR 1.33  （0.35 to 5.04） | 5 more per 1,000  (from 9 fewer to 56 more) | Very low  ⊕〇〇〇 | Critical |

Supplementary Table 2. Evidence profile (Including all RoB)

Reasons of downgrade: a) Sample size is smaller than optimal information size. In addition, 95% CI is wide. b) I2 value is high. c) Sample size is smaller than optimal information size. In addition, 95% CI is very wide. CI: Confidence interval; RR: Risk ratio; RoB: Risk of Bias.
